# Antibacterial Activities of Selected Pure Compounds Isolated from Gut Bacteria of Animals Living in Polluted Environments

**DOI:** 10.3390/antibiotics9040190

**Published:** 2020-04-17

**Authors:** Noor Akbar, Ruqaiyyah Siddiqui, Mazhar Iqbal, Naveed Ahmed Khan

**Affiliations:** 1 Department of Biological Sciences, School of Science and Technology, Sunway University, Bandar Sunway 47500, Malaysia; noormicrobiologist555@gmail.com; 2 Department of Biology, Chemistry and Environmental Sciences, College of Arts and Sciences, American University of Sharjah, Sharjah 26666, UAE; ruqaiyyah_s@hotmail.com; 3 National Institute for Biotechnology and Genetic Engineering, Faisalabad 44000, Pakistan; hamzamgondal@gmail.com

**Keywords:** antibiotic resistance, antibacterials, N-acyl homoserine lactones, Di-rhamnolipids, cytotoxicity

## Abstract

Antibiotic resistance is a global threat to public health, further accelerated by the misuse of antibiotics in humans and animals. Our recent studies have shown that gut bacteria of animals living in polluted environments are a potential source of antibacterials. Gut bacteria of cockroaches, water monitor lizards and the turtle exhibited molecules such as curcumenol, docosanedioic acid, N-acyl-homoserine lactone, L-homotyrosine and Di-rhamnolipids. Using purified compounds, assays were performed to determine their antibacterial properties using serial dilution method, cytotoxic effects using lactate dehydrogenase release, and cell viability using MTT assay. The results revealed that the purified compounds exhibited significant antibacterial activities (*p* < 0.05) against selected Gram-negative (*Pseudomonas aeruginosa*) and Gram-positive bacteria (*Streptococcus pyogenes*) with effective MIC_50_ and MIC_90_ at µg concentrations, and with minimal effects on human cells as observed from LDH and MTT assays. These findings are significant and provide a basis for the rational development of therapeutic antibacterials. Future studies are needed to determine in vivo effects of the identified molecules together with their mode of action, which could lead to the development of novel antibacterial(s).

## 1. Introduction

The emergence of antibiotic resistance is a public health crisis [1,2]. Several lines of evidence suggest that bacterial pathogens have evolved dramatic multi-drug resistance in recent years resulting in a major public health threat globally [1,2,3]. For example, *Pseudomonas aeruginosa* have emerged as a major cause of nosocomial infections, particularly in immuno-compromised patients [4]. In 2015, European Centers for Disease Prevention and Control (ECDC) reported that five strains out of every 100 invasive *P. aeruginosa* isolates were found to be resistant against all five antibacterial groups (piperacillin-tazobactam, fluoroquinolones, ceftazidime, aminoglycosides and carbapenems) under surveillance (EARS-Net) whereas, 13.7% were resistant to three antimicrobial groups available [5]. In the United States, multi-drug resistant *P. aeruginosa* causes 13% of severe health care-associated infections [5]. Similarly, in 2017 reports from World Health Organization (WHO) have shown that carbapenem-resistant *P. aeruginosa* was listed in the “critical” group [6]. Of the Gram-positive pathogens, *Streptococcus pyogenes* is responsible for more than 600 million infections annually [7]. *S. pyogenes* causes a wide range of conditions and diseases from minor skin and soft tissue infections to severe clinical manifestations [8]. Collectively, these findings suggest that novel antibacterials are needed to counter increasing bacterial resistance [9,10]. 

Microbial derived natural products possess a wide range of biological properties including antibacterial activities. Most antibacterial classes are derived from readily-available soil bacteria [10,11], but this resource has been mined for several decades [12]. In addition to soil, bacteria isolated from unusual environments can be a potential source of antibacterials. Recent studies have shown that gut bacteria of animals living in polluted environments are a potential source of antibacterials [12,13,14,15,16,17,18,19,20]. The microbiota associated with animal’s gastrointestinal tract (GIT) is a novel and fascinating area of research [21]. The gut microflora signifies the ecological community of microbes inhabiting the GI tract that ultimately influence development, immunity and physiology of all animals [22]. 

In this study, we used compounds isolated from the gut bacteria of cockroach and turtle. These compounds include (curcumenol and L-Homotyrosine) produced by *Bacillus subtilis*, (docosanedioic acid) produced by *E. coli*, and (N-tetradecanoyl homoserine lactone and Di-rhamnolipids) isolated from *P. aeruginosa*. These compounds were tested against selected Gram-positive and Gram-negative bacteria and human cell lines. Overall, the isolated compounds showed promising antibacterial activities against both Gram-positive and Gram-negative bacteria. 

## 2. Results

### 2.1. Curcumenol, L-Homotyrosine and Docosanedioic Acid Showed Significant Antibacterial Activities Against Gram-Negative and Gram-Positive Pathogenic Bacteria Tested

**Figure 1:**
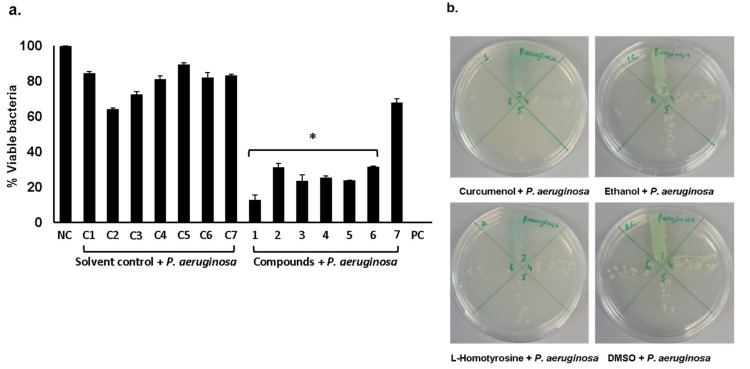
Antibacterial assays of pure compounds against *P. aeruginosa.* Briefly, 50 µg/mL of each compound was incubated with 1 × 10^6^ bacteria at 37 °C for 2 h. Next, cultures were serially diluted and plated onto agar plates. Following this incubation, viable bacterial colonies were counted and c.f.u. was recorded. (**a**) Bactericidal activities of curcamenol, L-homotyrosine and docosanedioic acid against *P. aeruginosa.* (**b**) Representative effects of compounds on *P. aeruginosa*. The data are expressed as the mean ±SE of several independent experiments performed in duplicate. *P*-values were determined by student’s T-test where (*) represents *P* < 0.05 using GraphPad Prism 8.0.2.

In our previous studies, we have isolated gut bacteria from animals/pests and tested their conditioned media (extracts) against multi-drug resistant Gram-negative and Gram-positive bacteria [9,15]. Bioactive molecules were identified (see Supplementary Figures S1–S5) [9,23] and out of which a few selected compounds were obtained from Sigma Aldrich and tested for their antibacterial properties. The results revealed that all three compounds exhibited significant bactericidal activities (Figure 1a,b) (*P* < 0.05, using student’s t-test, two-tailed distribution). Among all tested compounds, curcumenol showed exceptional bactericidal effects (Figure 1a) killing 87% bacteria. The minimum inhibitory concentrations (MIC_50_ and MIC_90_) for curcumenol, L-homotyrosine and docosanedioic acid against *P. aeruginosa* are shown in Table 1.

**Figure 2:**
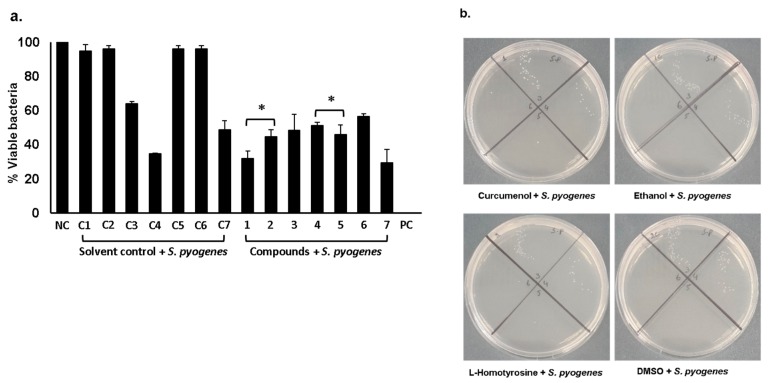
Antibacterial assays of pure compounds against *S. pyogenes.* Briefly, 1 × 10^6^ bacteria were exposed to 50 µg/mL of each compound at 37 °C for 2 h. After this, cultures were serially diluted, plated onto agar plates and plates were incubated overnight at 37 °C. Next day, bacterial c.f.u was determined by counting viable colonies. (**a**) Antibacterial activities of curcamenol, L-homotyrosine and docosanedioic acid against *S. pyogenes.* (**b**) Representative effects of compounds on *S. pyogenes*. *P*-values were determined by student’s T-test where (*) represents *P* < 0.05 using GraphPad Prism 8.0.2. The data are expressed as the mean ±SE of several independent experiments performed in duplicate.

When these compounds were tested against *S. pyogenes*, the results showed that among all the three tested compounds, curcumenol and L-homotyrosine exhibited significant bactericidal activities (Figure 2a,b) (*P* < 0.05). Curcumenol alone as well as in combination with L-homotyrosine and docosanedioic acid showed important antibacterial properties (Figure 2a) (*P* < 0.05). Docosanedioic alone and in combination with L-homotyrosine did not show antibacterial effects. The MIC values of these compounds against *S. pyogenes* are shown in Table 1.

### 2.2. Di-Rhamnolipids and N-Tetradecanoyl Homoserine Lactones Showed Promising Antibacterial Activities Against Gram-Positive and Gram-Negative Bacteria

**Figure 3:**
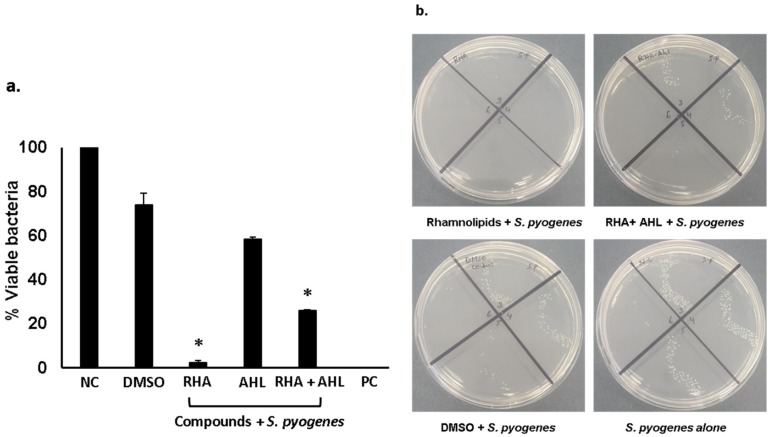
Antibacterial assays of pure compounds against *S. pyogenes.* Briefly, 1 × 10^6^ bacteria were incubated with pure compounds at 50 µg/mL for 2 h at 37 °C. Next, cultures were diluted, plated onto agar plates and plates were incubated overnight at 37 °C. Following this incubation, bacterial c.f.u was determined by counting viable colonies. (**a**) bactericidal and (**b**) bacteriostatic activities of rhamnolipids and *N*-acyl homoserine lactones against *S. pyogenes. P*-values were determined by student’s T-test where (*) represents *P* < 0.05 using GraphPad Prism 8.0.2. The data are expressed as the mean ± SE of several independent experiments performed in duplicate.

**Figure 4:**
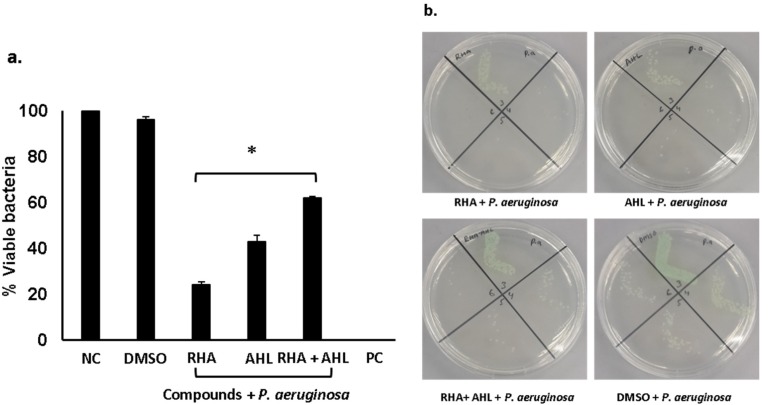
Antibacterial assays of pure compounds against *P. aeruginosa.* Briefly, pure compounds at 50 µg/mL were incubated with 1 × 10^6^ bacteria for 2 h at 37 °C. Next, cultures were diluted and plated onto agar plates. The plates were incubated at 37 °C for overnight. After this incubation, viable bacterial colonies were counted to determine, (**a**) bactericidal and (**b**) bacteriostatic activities of rhamnolipids and *N*-acyl homoserine lactones against *P. aeruginosa.* The data are expressed as the mean ± SE of several independent experiments performed in duplicate. *P*-values were determined by student’s T-test where (*) represents *P* < 0.05 using GraphPad Prism software 8.0.2.

Di-rhamnolipids and *N*-tetradecanoyl homoserine (AHL) lactone (see supplementary Figure S1) from *P. aeruginosa* isolated from turtle’s gut bacteria and were tested for their antibacterial activities [9]. The results revealed that rhamnolipids showed exceptional bactericidal activity (>90%) against *S. pyogenes* (Figure 3) (*P* < 0.05). When tested in combination with AHL, synergistic effects were observed and significant antibacterial activity against *S. pyogenes* was revealed (Figure 3) (*P* < 0.05). When Di-rhamnolipids were tested against *P. aeruginosa,* rhamnolipids alone as well as in combination showed important bactericidal activities (Figure 4) (*P* < 0.05). Similarly, AHL were tested against both *S. pyogenes* and *P. aeruginosa*. AHL alone failed to show antibacterial activity against *S. pyogenes* whereas combined with RHA showed notable bactericidal activity (Figure 3) (*P* < 0.05). Unlike against *P. aeruginosa,* AHL showed promising antibacterial effects alone as well as in combination with rhamnolipids (Figure 4) (*P* < 0.05). The MIC_50_ and MIC_90_ for Di-rhamnolipids and AHLs against *P. aeruginosa* and *S. pyogenes* are shown in Table 1.

### 2.3. Pure Compounds Showed Minimal Cytotoxic Effects against Human Cell Lines

**Figure 5:**
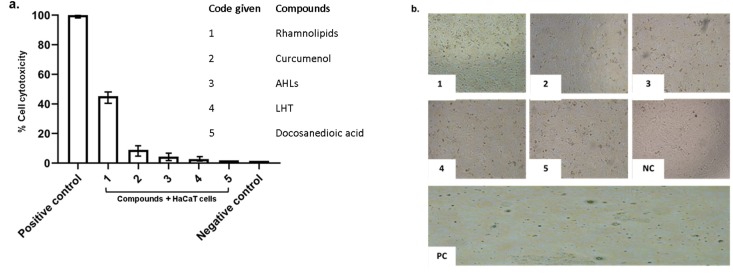
In vitro cytotoxic effects of compounds against HaCaT cell lines. Briefly, pure compounds were incubated with HaCaT cells monolayer in a 96 well plate at 37 °C for 24 h in the presence of 5% CO_2_ and humidified conditions. Following day, LDH released by cells was measured at 490 nm and results were recorded. (**a**) Pure compounds tested were non-toxic against HaCaT cells. (**b**) Representative images and compounds effects incubated with cell monolayer.

**Figure 6:**
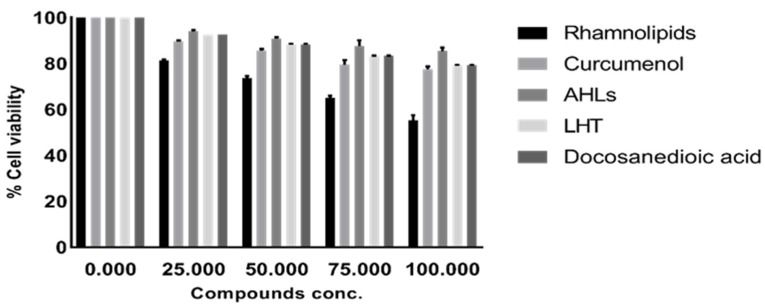
Cytotoxic effects of compounds isolated from gut bacteria in a concentration dependent manner on HaCaT cell viability using MTT assays. Briefly, overnight grown confluent HaCaT cells monolayer was exposed to different concentration of the compounds in a 96-well plate and plate was incubated at 37 °C for 24 h in the presence of 5% CO_2_ and humidified conditions. HaCaT cells alone was taken as negative control. All data are expressed as the mean ± standard error of three independent experiments performed in duplicates. Data was analyzed using GraphPad prism software version 8.0.2.

Lactate dehydrogenase assays were performed to determine the cytotoxic effects of pure compounds, against HaCaT cell lines. The results showed that all pure compounds tested showed minimal/ or no cytotoxic effects against human cells lines except Di-rhamnolipids that showed 45% cytotoxicity (Figure 5). Moreover, the compounds were tested at various concentrations to determine their effects on cell viability. The results revealed that among all compounds tested, Di-rhamnolipids showed cytotoxicity at 50 µg/mL and 100 µg/mL against HaCaT cell lines (Figure 6). All other compounds exhibited minimal cytotoxicity which is considered as non-toxic according to ISO 10993-5 [9].

## 3. Discussion

Antibiotic resistance is a major threat to public health [15,24,25,26]. Most of the antibacterial agents available commercially are derived from plants, and microbes, especially bacteria [27]. The producer’s species are utilizing these molecules as competitive weapons against other bacteria, parasites and pathogenic fungi [28]. Natural products especially bacterial secondary metabolites have been exploited successfully to target pathogenic microbes [15,29]. For example, Rahayu et al., [30] isolated curcumenol from the *Curcuma aeruginosa* plant rhizomes with significant antibacterial activity against Gram-negative (*Salmonella typhi* and *E. coli*) bacteria. Similarly, Diastuti et al., [31] isolated terpenoids and sesquiterpenes from *Curcuma heyneana* rhizomes that showed antibacterial activities. Kacem et al., [32] tested hydrodistilled oil contained curcumenol as component obtained from plant i.e., *Genista quadriflora* against *S. aureurs.* Crude extracts showed significant antibacteria activities against *S. aureurs*. In these studies, curcumenol was isolated either from plant rhizomes or oil but here in this study we identified curcumenol from *Bacillus subtilis* isolated from the gut of cockroaches (*Blaptica dubia*) that showed antibacterial activities against both Gram-negative (*P. aeruginosa*) and Gram-positive (*S. pyogenes*) bacteria. The bactericidal activity of curcumenol was higher against *P. aeruginosa* as compared to *S. pyogenes.*

*Bacillus subtilis* also produced L-homotyrosine. This compound showed broad-spectrum antibacterial activities against both *S. pyogenes* and *P. aeruginosa*. L-homotyrosine exhibited antibacterial activities against *P. aeruginosa* and its possible mode of action is inhibition of 4-hydroxyphenylpyruvate dioxygenase enzymes of *P. aeruginosa* [33]. This compounds also showed bactericidal activities against *Staphylococcus aureus* [34]. L-homotyrosine has been shown to possess antifungal activity against *Candida albicans* and *C. glabrata* by inhibiting β-1,3-glucan synthesis [35]. Docosanedioic acid was identified from *E. coli* isolated from cockroach (*Gromphadorhina portentosa*) gut. Docosanedioic acid showed antibacterial activities against *P. aeruginosa* but failed to show bactericidal activity against *S. pyogenes.* This compound possesses several biological activities reported previously such as anti-HIV, anti-inflammatory, anti-cancer and antifungal activities against *A. fumigatus*, *C. neoformans* and *C. albicans* [36]. We tested Docosanedioic acid for their antibacterial activities and revealed notable bactericidal effects against Gram-negative *P. aeruginosa*.

Similarly, *P. aeruginosa* isolated from turtle gut produced strong quorum sensing molecules i.e., N-acyl homoserine lactones (AHL) (*N*-Tetradecanoyl homoserine) and biological biosurfactant i.e., Di-rhamnolipids [9]. The antibacterial assays of these molecules showed that AHL showed promising results against *P. aeruginosa* while no antibacterial activity was observed against *S. pyogenes*. Rhamnolipids showed exceptional bactericidal activity against *S. pyogenes* and *P. aeruginosa*. Similar molecules have been isolated from *P. aeruginosa* isolated from different sources. For example, Patel et al., [37] screened several Gram-negative bacteria including *P. aeruginosa* that produced AHLs involved in cell-to-cell communication. In another study, Kušar et al., [38] quantified AHLs from clinical samples of *P. aeruginosa* isolated from dog with otitis externa. Rhamnolipids isolated from *P. aeruginosa* OBP1 significantly alters the cell membrane of *K. pneumoniae* and *S. aureus* and showed significant antibacterial activities [39]. Dusane et al., [40] tested rhamnolipids against *Bacillus pumilus* and results showed dislodging of biofilm formed by *B. pumilus* at very low concentrations, signifying their role in eradicating pre-formed biofilms [40].

In summary, these findings suggest that gut bacteria of animals living in polluted environments are a potential source of antibacterials. Gut bacteria of cockroaches, water monitor lizards and turtle exhibited molecules such as curcumenol, docosanedioic acid, N-acyl-homoserine lactone, L-homotyrosine and Di-rhamnolipids with significant antibacterial activities and minimal effects on human cells. These findings are significant and provide a basis for the rational development of therapeutic antibacterials. Future studies are needed to determine in vivo effects of the identified molecules together with their mode of action, which could lead to the development of novel antibacterial(s).

## 4. Materials and Methods

### 4.1. Bacterial Cultures

Bacterial species used in this study include multi-drug resistant *Pseudomonas aeruginosa* (ATCC 10145) and *Streptococcus pyogenes* (ATCC 49399) and grown overnight at 37 °C and maintained aerobically prior to experimentations as previously described [23]. 

### 4.2. Preparation of Compounds Stock Solution

The purified compounds identified from animal’s gut bacteria were dissolved in their respective solvents. Di-rhamnolipids, N-tetradecanoyl homoserine lactones and L-Homotyrosine were dissolved in Di-methyl sulfoxide (DMSO), Curcumenol was dissolved in ethanol and docosanedioic acid was dissolved in n-hexane. All compounds were isolated and identified using high performance liquid chromatography and mass spectrometry (See supplementary Figure S1) [9,15]. All the required stock solutions were prepared and stored at their optimum temperature before testing for their antibacterial and cytotoxic activities.

### 4.3. Evaluation of Pure Compounds for Antibacterial Properties

Antibacterial assays were performed as described previously [9] with some modifications. Pure compounds at final concentration of 50 µg/mL (Table 2) were incubated with 1 × 10^6^ bacterial inoculums for 2 h at 37 °C. Next, cultures were serially diluted and plated on agar plates. Plates were incubated overnight at 37 °C and viable bacterial colonies were counted. In some of the experiments, the compounds were evaluated at various concentrations (100, 50, 25, 12.5 and 6.25 µg/mL) to determine minimum inhibitory concentrations i.e., MIC_50_ and MIC_90_ against *S. pyogenes* and *P. aeruginosa*. For MIC, 1 × 10^5^ bacteria per well for controls and pure compounds were tested. Bacteria grown in Muller Hinton broth (MHB) alone and different solvents used for compounds preparation were used as negative control, while gentamicin was used as a positive control.

### 4.4. In Vitro Cytotoxicity Assays

Cell cytotoxicity assays were performed using MTT (3-(4,5-dimethylthiazol-2-Yl)-2,5-diphenyltetrazolium bromide) assays [9] and lactate dehydrogenase (LDH) assays [41] to determine the effects of pure compounds against human cell lines. Briefly, for LDH assays, confluent HaCaT cell monolayers in a 96-well plate was challenged with 50 µg/mL of pure compounds and plates were incubated for 24 h at 37 °C in the presence of 5% CO_2_ and humidified conditions. Next, the supernatant was mixed with an equal volume of LDH kit reagents and data analysis was performed spectrophotometrically at 490 nm. For MTT assays, pure compounds were incubated at various concentrations with overnight grown confluent monolayers of human cells and plates were incubated at 37 °C for overnight at 95% humidity and 5% CO_2_. After this incubation, 10 µL of MTT solution was added to each well and plates was incubated for 3–4 h at 37 °C. The MTT solution was withdrawn carefully and 100 µL of DMSO was added to dissolve the crystals formed by viable cells. The plates were incubated for 15 min at 37 °C and the absorbance determined at 540 nm immediately.

### 4.5. Statistical Analysis

Student T-test was used to analyze data for antibacterial assays using GraphPad Prism 8.0.2 software. LCMS data analysis (Supplementary data was analyzed using Agilent MassHunter Qualitative Analysis B.05.00 and Thermo Scientific Xcalibur software). The data are expressed as the mean ± standard error of three independent experiments carried out in duplicate. *P*-value < 0.05 was defined as data is significant.

### 4.6. Ethical Approval and Consent to Participate

This article does not contain any studies using humans and animals. We also confirm that all experiments were performed in accordance with relevant guidelines and regulations.

## 5. Conclusions

In summary, here we tested selected pure compounds isolated from animal gut bacteria for their bactericidal activities against Gram-positive and Gram-negative bacteria. Most of the compounds showed significant antibacterial activities with minimum and/or no cytotoxicity with the exception of Di-rhamnolipids that exhibited minimal cytotoxic activities against human cells. These outcomes are important and should lead to the rational development of novel curative antibacterial drugs. In future, such bioactive molecules possessing antibacterial activities could be tested in vivo using mouse animal model.

## Figures and Tables

**Table 1:** MIC_50_ and MIC_90_ values of pure compounds against Gram-positive and Gram-negative bacteria.

S. No	Compounds	*S. Pyogenes*		*P. Aeruginosa*	
		MIC_50_	MIC_90_	MIC_50_	MIC_90_
1	Rhamnolipids	34.6 µg/mL	98.36 µg/mL	44. 06 µg/mL	86.87 µg/mL
2	AHL	74.1 µg/mL	140.06 µg/mL	44.53 µg/mL	87.73 µg/mL
3	Curcumenol	82.26 µg/mL	156 µg/mL	82.96 µg/mL	165.69 µg/mL
4	L-Homotyrosine	73.27 µg/mL	141.45 µg/mL	51.39 µg/mL	94.25 µg/mL
5	Docosanedioic acid	111.1 µg/mL	204.76 µg/mL	220.8 µg/mL	405.48 µg/mL

AHL: N-acyl homoserine lactone.

**Table 2:** Pure compounds identified from animal’s gut bacteria used in this study.

Codes Given	Compounds/Combinations and Their Concentrations
1	Curcumenol (50 µg/mL)
2	L-Homotyrosine (50 µg/mL)
3	Docosanedioic acid (50 µg/mL)
4	Curcumenol (25 µg/mL) + L-Homotyrosine (25 µg/mL)
5	Curcumenol (25 µg/mL) + Docosanedioic acid (25 µg/mL)
6	L-Homotyrosine (25 µg/mL) + Docosanedioic acid (25 µg/mL)
7	Curcumenol (16.67 µg/mL) + L-Homotyrosine (16.67 µg/mL) + Docosanedioic acid (16.67 µg/mL)
C1	Ethanol
C2	DMSO
C3	n- hexane
C4	Ethanol + DMSO + n- hexane
C5	Ethanol + DMSO
C6	Ethanol + n-hexane
C7	DMSO + n- hexane
NC	Bacteria alone
PC	Bacteria + Gentamicin
RHA	Rhamnolipids (50 µg/mL)
AHL	N-Tetradecanonyl homoserine lactone (50 µg/mL)
RHA + AHL	Rhamnolipids (25 µg/mL+ *N*-Tetradecanonyl homoserine lactone (25 µg/mL)

## Supplementary Materials

The following are available online at https://www.mdpi.com/2079-6382/9/4/190/s1, Figure S1: Curcumenol, Figure S2: L-Homotyrosine; Figure S3: Docosanedioic acid; Figure S4: N-Tetradecanoyl-homoserine lactone; Figure S5 Di-Rhamnolipids.
