# Supplementary material for: Antibacterial Activities of Selected Pure Compounds Isolated from Gut Bacteria of Animals Living in Polluted Environments
Source: Antibiotics (Basel). 2020 Apr 17;9(4):190. doi: 10.3390/antibiotics9040190 (PMC7235713; doi:10.3390/antibiotics9040190)

<u>**Supplementary Data**</u>

Supplementary Figure S1-S3

S1. Curcumenol

Compound Chromatograms

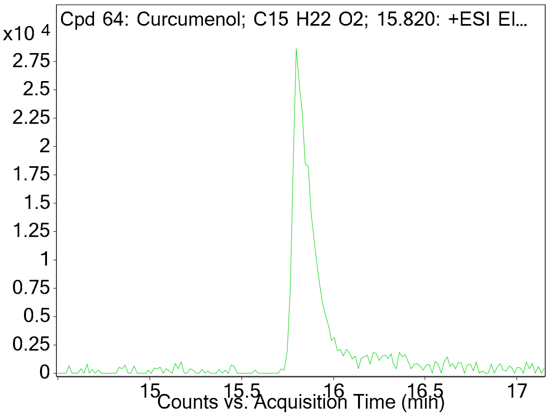

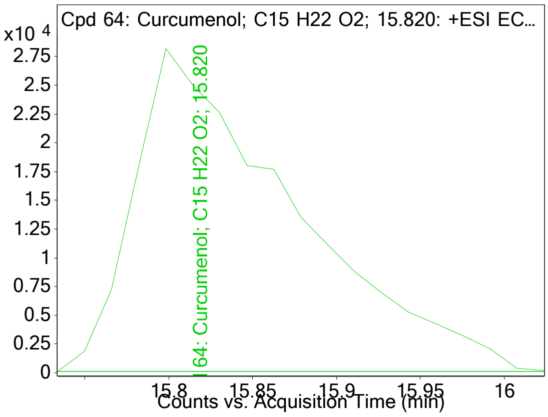

MFE MS Spectrum

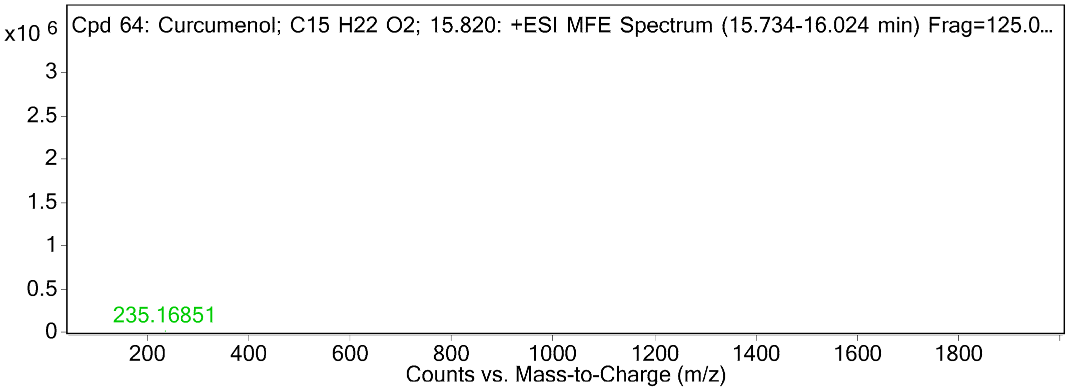

MFE MS Zoomed Spectrum

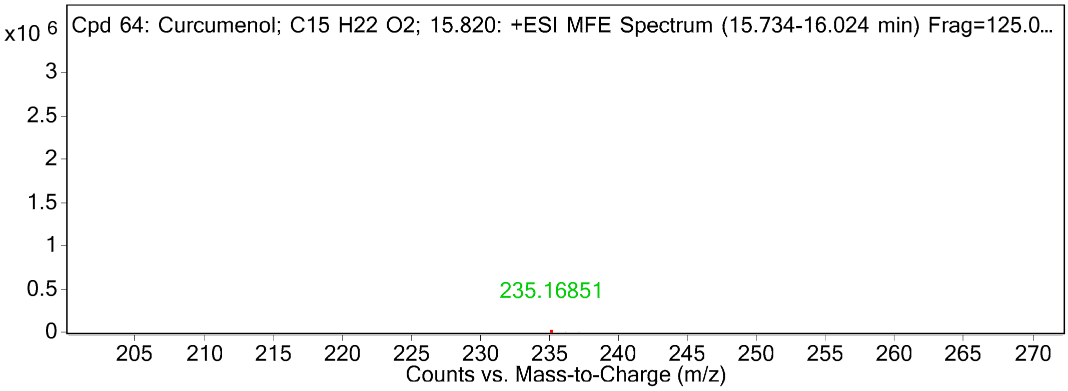

MS Spectrum Peak List

m/z	**z**	**Abund**	**Formula**	**Ion**
235.16851	1	23410	C15 H23 O2	$(M+H)^+$
236.17177	1	4087.1	C15 H23 O2	$(M+H)^+$
237.17494	1	851.33	C15 H23 O2	$(M+H)^+$

Curcumenol Structure

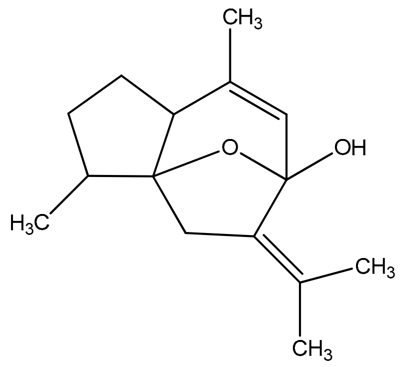

S2. L-Homotyrosine

Compound Chromatograms

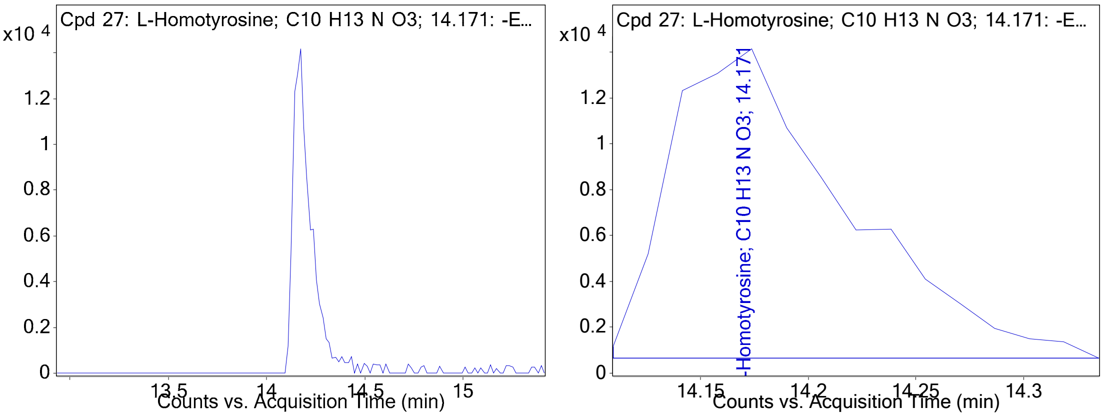

MFE MS Spectrum

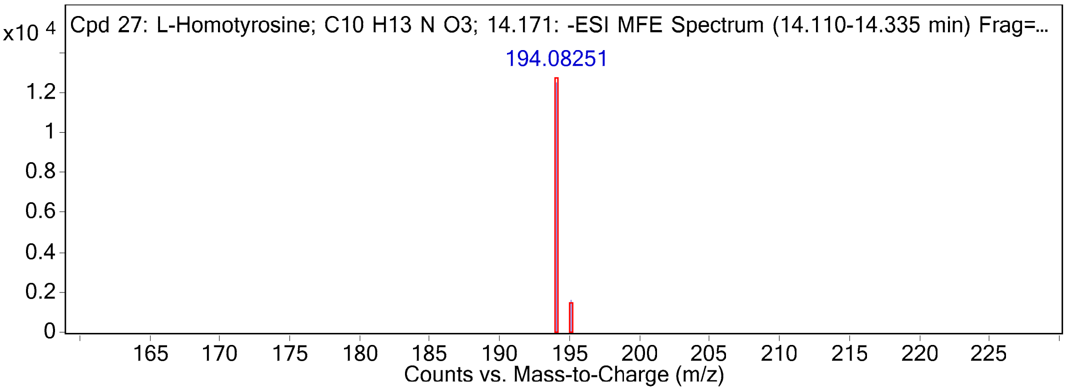

MFE MS Zoomed Spectrum

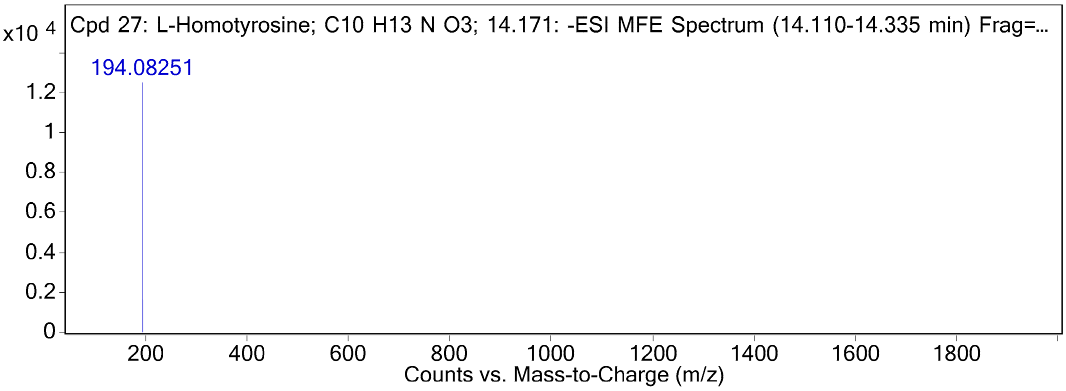

MS Spectrum Peak List

m/z	z	Abundance	Formula	Ion
194.08251	-1	12505.16	C10 H12 N O3	(M-H)$^-$
195.08562	-1	1653.06	C10 H12 N O3	(M-H$^{)-}$

L-Homotyrosine Structure

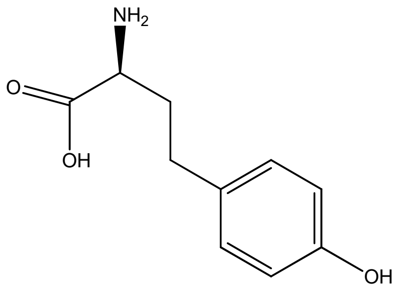

S3. Docosanedioic acid

Compound Chromatograms

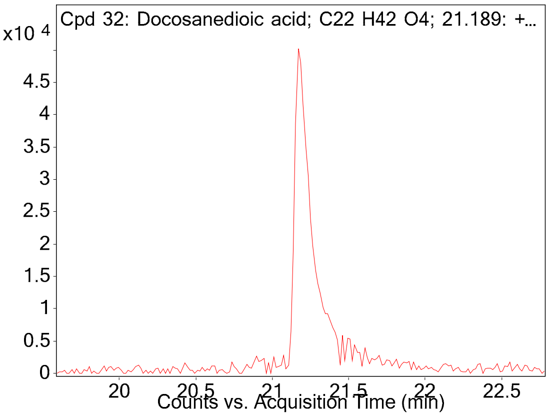

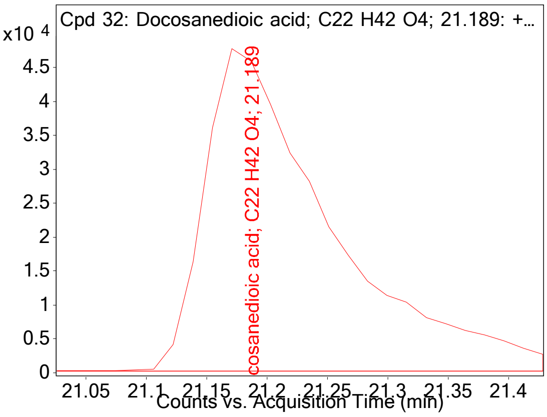

MFE MS Spectrum

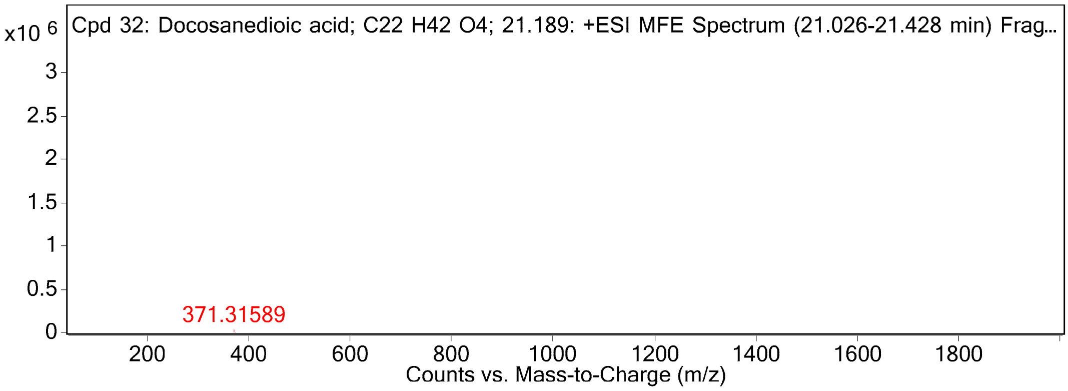

MFE MS Zoomed Spectrum

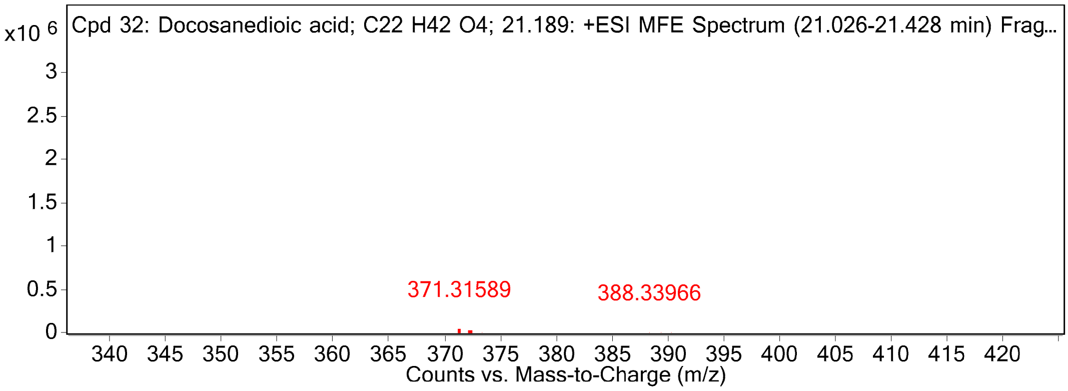

MS Spectrum Peak List

m/z	z	Abund	Formula	Ion
371.31589	1	35416.75	C22 H43 O4	$(M+H)^+$
372.31933	1	8814.32	C22 H43 O4	$(M+H)^+$
373.31529	1	1912.73	C22 H43 O4	$(M+H)^+$
388.33966	1	1216.57		$(M+NH4)^+$
389.34669	1	371.23		$(M+NH4)^+$
390.34676	1	395.32		$(M+NH4)^+$

Docosanedioic acid Structure

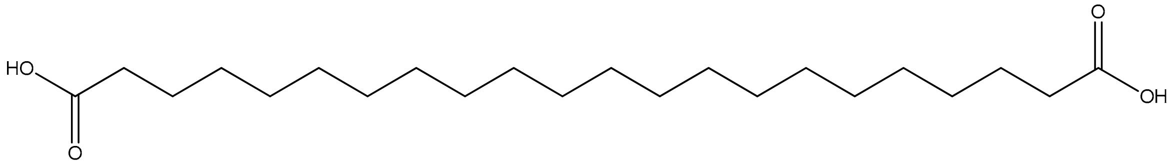

Supplement: Supplementary file 1 [file antibiotics-09-00190-s001.zip › Supplementary Fig S1-3.pdf]
