# Supplementary figures and images for: Antibacterial Activities of Selected Pure Compounds Isolated from Gut Bacteria of Animals Living in Polluted Environments
Source: Antibiotics (Basel). 2020 Apr 17;9(4):190. doi: 10.3390/antibiotics9040190 (PMC7235713; doi:10.3390/antibiotics9040190)

## Supplementary Figure S4-S5

### S4. *N*-Tetradecanoyl-homoserine lactone

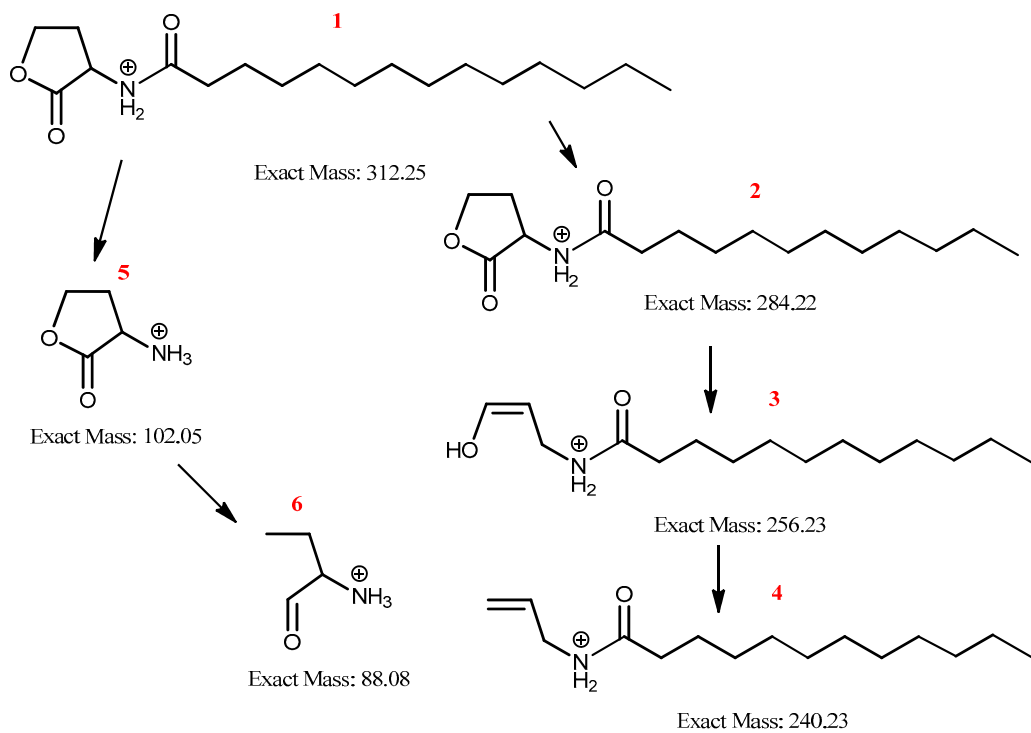

## S5. Di-Rhamnolipids

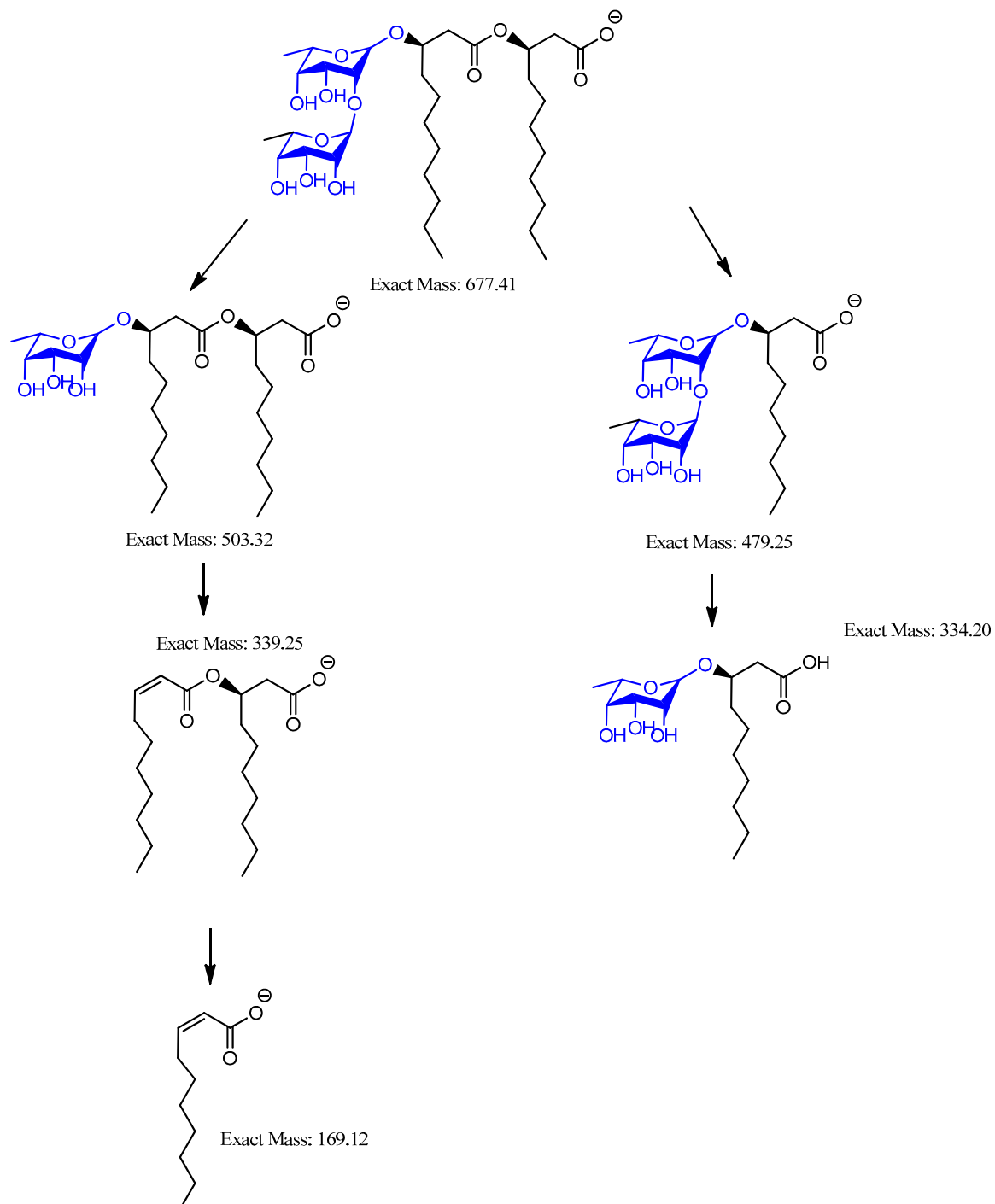

Supplement: Supplementary file 1 [file antibiotics-09-00190-s001.zip › Supplementary Fig S4-S5.pdf]
